# Blood Pressure Control Has Improved in People with and without Type 2 Diabetes but Remains Suboptimal: A Longitudinal Study Based on the German DIAB-CORE Consortium

**DOI:** 10.1371/journal.pone.0133493

**Published:** 2015-07-29

**Authors:** Ina-Maria Rückert, Jens Baumert, Michaela Schunk, Rolf Holle, Sabine Schipf, Henry Völzke, Alexander Kluttig, Karin-Halina Greiser, Teresa Tamayo, Wolfgang Rathmann, Christa Meisinger

**Affiliations:** 1 Institute of Epidemiology II, Helmholtz Zentrum München - German Research Center for Environmental Health (GmbH), Neuherberg, Germany; 2 German Center for Diabetes Research (DZD e.V.), Partner Neuherberg, Ingolstädter Landstraße 1, 85764 Neuherberg, Germany; 3 Institute of Health Economics and Health Care Management, Helmholtz Zentrum München - German Research Center for Environmental Health (GmbH), Neuherberg, Germany; 4 Institute for Community Medicine, University Medicine Greifswald, Greifswald, Germany; 5 DZHK-German Centre for Cardiovascular Research, partner site Greifswald, Greifswald, Germany; 6 Institute of Medical Epidemiology, Biostatistics and Informatics, Martin-Luther-University Halle-Wittenberg, Halle (Saale), Germany; 7 German Cancer Research Centre, Division of Cancer Epidemiology, Heidelberg, Germany; 8 Institute of Biometrics and Epidemiology, German Diabetes Center, Leibniz Center for Diabetes Research at Heinrich-Heine-University Düsseldorf, Düsseldorf, Germany; 9 MONICA/KORA Myocardial Infarction Registry, Central Hospital of Augsburg, Augsburg, Germany; Medical University Innsbruck, AUSTRIA

## Abstract

**Background:**

Hypertension is a very common comorbidity and major risk factor for cardiovascular complications, especially in people with Type 2 Diabetes (T2D). Nevertheless, studies in the past have shown that blood pressure is often insufficiently controlled in medical practice. For the DIAB-CARE study, we used longitudinal data based on the German DIAB-CORE Consortium to assess whether health care regarding hypertension has improved during the last decade in our participants.

**Methods:**

Data of the three regional population-based studies CARLA (baseline 2002-2006 and follow-up 2007-2010), KORA (baseline 1999-2001 and follow-up 2006-2008) and SHIP (baseline 1997-2001 and follow-up 2002-2006) were pooled. Stratified by T2D status we analysed changes in frequencies, degrees of awareness, treatment and control. Linear mixed models were conducted to assess the influence of sex, age, study, and T2D status on changes of systolic blood pressure between the baseline and follow-up examinations (mean observation time 5.7 years). We included 4,683 participants aged 45 to 74 years with complete data and accounted for 1,256 participants who were lost to follow-up by inverse probability weighting.

**Results:**

Mean systolic blood pressure decreased in all groups from baseline to follow-up (e.g. – 8.5 mmHg in those with incident T2D). Pulse pressure (PP) was markedly higher in persons with T2D than in persons without T2D (64.14 mmHg in prevalent T2D compared to 52.87 mmHg in non-T2D at baseline) and did not change much between the two examinations. Awareness, treatment and control increased considerably in all subgroups however, the percentage of those with insufficiently controlled hypertension remained high (at about 50% of those with hypertension) especially in prevalent T2D. Particularly elderly people with T2D often had both, high blood pressure ≥140/90 mmHg and a PP of ≥60 mmHg. Blood pressure in men had improved more than in women at follow-up, however, men still had higher mean SBP than women at follow-up.

**Conclusion:**

Blood pressure management has developed positively during past years in Germany. While hypertension prevalence, awareness and treatment were substantially higher in participants with T2D than in those without T2D at follow-up, hypertension control was achieved only in about half the number of people in each T2D group leaving much room for further improvement.

## Introduction

### Background

The UK Hypertension in Diabetes Study, a sub-study of the UK Prospective Diabetes Study, was the first to reveal that hypertension is very common in people with T2D and that treatment and control are safe and can have great beneficial effects in terms of a reduction of cardiovascular morbidity and mortality [1]. These findings date back to 1998 and thus are relatively novel. Since then hypertension and T2D have been studied more intensively, and numerous guidelines by international expert committees have been devised in order to instruct and support physicians in the treatment of patients with hypertension and T2D (e.g. [2, 3]).

However, it has also turned out, that blood pressure control remains a complicated and problematic issue in Germany and throughout the world [4, 5]. People with hypertension are often unaware of their disease, they appear to underestimate the clinical significance of high blood pressure, and/or do not achieve their treatment goals because of various reasons (e.g. [6, 7]). Moreover, there has been a controversial discussion about which blood pressure parameter actually is most important for risk prediction and should be focussed on in clinical practise. Systolic blood pressure (SBP) rises with advancing age, while diastolic blood pressure (DBP) tends to drop, which is a sign of an increasing stiffness of the large arteries [8]. Pulse pressure (PP) unites both components and is defined as the difference of SBP and DBP. A deviation of more than around 60 mmHg is considered as a risk factor for cardiovascular events and mortality (e.g. [9, 10]) and is found more often in older people due to the shifts of SBP and DBP in association with age. Most analyses, however, point to the importance of SBP [11, 12]. The guidelines and cut-points to define hypertension have also changed over time with additional medical insights. So far, it is not clear, whether people at higher risk of cardiovascular events really benefit from blood pressure values of <130/80 mmHg (as recommended by the WHO/ISH in 2003 [13]) compared to the more conservative categorization of <140/90 mmHg. The American Diabetes Association, for example, currently recommends a target of <140/80 mmHg for people with diabetes [2]. Yet, the risk for cardiovascular disease appears to rise continuously starting with “normal” and “high normal” readings [14, 15].

During the study period of the DIAB-CORE studies, national German guidelines on the therapy of diabetes recommended an ideal blood pressure of <130/85 mmHg, values between 130/85 mmHg and 140/90 mmHg were considered as elevated and values >140/90 mmHg as high risk conditions. In due consideration of the patient’s age, general health and further comorbidities, values >130/85 mmHg could be acceptable in individual cases [16]. The newest update from 2014 states <140/80 mmHg as therapeutic aim [17]. Since specific differing values for elderly people were not mentioned in these guidelines, we used a conservative cut-point of 140/90 mmHg for all participants in the current analyses.

By all means, hypertension and T2D are common conditions in developed societies and the prevalences have been increasing noticeably in developing countries [18]. Cardiovascular risk factors used to be absent in traditional folks e.g. the Solomon Islands societies [19] and are very variable in different ethnicities and with changing lifestyles [20, 21], which implies that improved health behaviour and treatment should be effective in bringing the hypertension epidemic under control (e.g. by improving dietary factors [22]). The current analysis was intended to contribute information on the development of hypertension frequency, awareness, treatment and control in people with and without T2D in Germany.

### Objectives

Using baseline data of the German DIAB-CORE Consortium, we described insufficient blood pressure control and medication in persons with T2D in two previous publications [23, 24]. The successor study DIAB-CARE included follow-up data of three DIAB-CORE studies and aimed at the examination of changes in blood pressure control in participants with and without T2D during approximately ten years (study period 1997–2010). We looked at different blood pressure parameters and applied descriptive methods and multivariable adjusted regression models.

## Materials and Methods

### Study design and setting

The current analysis included data from three population-based studies in Germany that are part of the DIAB-CORE Consortium (from north to south): the Study of Health in Pomerania (**SHIP**, Greifswald), the Cardiovascular Disease, Living and Ageing (**CARLA**, Halle (Saale)) Study, and the Cooperative Health Research in the Region of Augsburg (**KORA**, Augsburg) Study, see Table 1. Baseline examinations of the studies were conducted between 1997 and 2006, follow-up examinations between 2002 and 2010. Similar instruments, questionnaires and medical measurements were used to assess participants’ characteristics. Detailed descriptions of study designs, samples and procedures are available elsewhere [25–27]. The investigations were carried out in accordance with the Declaration of Helsinki, including written informed consent of all participants. All study methods were approved by the Ethics Committee of the Medical Faculty of the Martin-Luther-University Halle-Wittenberg and by the State Data Privacy Commissioner of Saxony-Anhalt (CARLA), the Ethics Committee of the Bavarian Medical Association (KORA) and the Medical Ethics Committee of the University of Greifswald (SHIP). Primary study data of interest were pooled and frequencies compared.

**Table 1:** Studies included in the pooled sample (45–74 years), N = 5,939.

	SHIP^a^	CARLA^b^	KORA^c^	Total
**Study periods:** ^●^BL/^●●^FUP	1997–2001/2002–2006	2002–2006/2007–2010	1999–2001/2006–2008	1997–2006/2002–2010
**Response at baseline (%)**	69	64	67	
**Response at follow-up (%)**	84	86	80	
**Mean observation time** years(SD)	5.2 (0.6)	4.0 (0.3)	7.1 (0.2)	5.7 (1.3)
**N** (%)	2,219 (37.4)	1,356 (22.8)	2,364 (39.8)	5,939 (100.0)
**Women** %	50.4	47.3	50.7	49.8
**Age BL** mean (SD)	58.9 (8.5)	60.2 (7.9)	58.7 (8.6)	59.1 (8.4)
**Age FUP** mean (SD)	64.1 (8.5)	64.3 (7.9)	65.8 (8.6)	64.8 (8.5)
**T2D** * **BL** %	11.5	13.1	6.1	9.7
**T2D** * **FUP** %	17.3	18.5	12.2	15.6
**Hypertension** ** **BL %**	65.7	71.5	49.2	60.4
**Hypertension** ** **FUP %**	67.0	73.9	54.8	64.5

^a^ **SHIP** (Survey S-0/S1): Study of Health in Pomerania—North-east Germany (West Pomerania)

^b^ **CARLA**: Cardiovascular Disease, Living and Ageing in Halle—East Germany (Halle)

^c^ **KORA** (Survey S4/F4): Cooperative Health Research in the Region of Augsburg—South Germany (Augsburg region)

^●^BL: Baseline

^●●^FUP: Follow-Up

*Self-reported diabetes or self-reported use of anti-diabetic medication

**Blood pressure ≥140/90 mmHg or using anti-hypertensive medication

### Variables

#### Age

Participants were classified in three age groups ranging from 45 to 54, 55 to 64 and 65 to 74 years at baseline.

#### Type 2 diabetes

T2D was defined based on self-report or self-reported intake of oral anti-diabetic agents, insulin or a combination of both. Some studies lacked information on diabetes type. Thus, in order to exclude participants who probably had type 1 diabetes, self-reported age at diagnosis of diabetes was used, and only those patients with an age at diagnosis of > 30 years were included in the T2D group. T2D status with respect to baseline and follow-up examinations was defined in three groups: i) no T2D at baseline, no T2D at follow-up, ii) no T2D at baseline, T2D at follow-up (incident), iii) T2D at baseline, T2D at follow-up (prevalent).

#### Hypertension

In all three studies blood pressure measurements were carried out according to the same study protocol by trained and certified personnel with an automated oscillometric device (HEM-705CP, Omron Corporation, Tokyo, Japan). The measurements were taken after a rest period of at least five minutes in a sitting position and repeated three times at an interval of three minutes.

Hypertension was defined using the mean of the second and third blood pressure readings with SBP ≥140 mmHg and/or DBP ≥90 mmHg, or intake of anti-hypertensive medication in participants with a physician’s diagnosis of hypertension (“awareness”). Participants with hypertension were categorized into one of the following four subgroups: (1) aware (with physician’ diagnosis) and controlled treated to target levels of < 140/90 mmHg, (2) aware and treated, but not reaching target blood pressure values of < 140/90 mmHg, i.e. uncontrolled treated, (3) aware, but not treated, (4) unaware of hypertension. Thus, “awareness” of hypertension applied to participants in categories 1, 2 and 3, “treatment” applied to those in categories 1 and 2 and “control” to those in category 1.

The follow-up variable was delineated accordingly. However, the items in the questionnaires were not absolutely identical in the studies: in the KORA study, the question aimed at any discovery of hypertension irrespective of the kind of diagnosis (i.e. at a physician’s office, a pharmacy or at home). Therefore, the frequency in KORA may be somewhat higher than in the other two studies. The SHIP study asked for a physician’s diagnosis of hypertension during the last 5 years, and the CARLA study inquired about a physician’s diagnosis during the time that had passed since the baseline examination. We calculated the frequency of hypertension ‘ever’ for all follow-up examinations by including the information from the baseline examinations.

#### Pulse Pressure (PP)

PP was calculated by the subtraction of diastolic blood pressure from systolic blood pressure. A PP of greater than 50–65 mmHg is regarded to be unfavourable in the literature (e.g. [10, 28]). We chose a cutting point of ≥60 mmHg to define high PP.

#### Anti-hypertensive medication

All study participants were asked to bring original packaging of their medications used during the last seven days to the examinations at baseline and follow-up. The variable “anti-hypertensive medication” included prescription of medication belonging to the ATC subgroups C02 (antihypertensives), C03 (diuretics), C07 (beta blocking agents), C08 (calcium channel blockers) and C09 (agents reacting on the renin-angiotensin system), that were allowed for the treatment of chronic hypertension (e.g. as opposed to a one-time treatment of an acute hypertensive crisis) according to the German Hypertension League and/or the package inserts. Herbal (i.e. leaves of the stinging nettle, garlic, dandelion or others) and homeopathic preparations were excluded.

### Participants

For our current analyses, the pooled baseline data set consisted of 6,218 participants of German nationality aged 45 to 74 years at baseline examination. Of those, 4,901 (78.8%) also attended the follow-up examinations. Persons with missing data in one of the blood pressure variables (measurement (N (baseline) = 11, N (follow-up) = 19), physician’s diagnosis (N (baseline) = 42, N (follow-up) = 30) and/or intake of anti-hypertensive medication (N (baseline) = 11, N (follow-up) = 5) or missing information on T2D status (N (baseline) = 105, N (follow-up) = 139) were excluded. Thus, 4,683 participants with complete baseline and follow-up data on blood pressure and T2D comprised the complete case population of our analyses.

To account for the 1,256 baseline participants with complete baseline data on blood pressure and T2D who did not take part in the follow-up examinations, we calculated a weighted data set, regarding study, sex, age and T2D status. Persons who were lost to follow-up tended to be older, were more often male, suffered more often from T2D and had had a myocardial infarction or stroke more often than their counterparts. The weighted dataset thus consisted of 5,939 entries based on the population of 4,683 participants with complete baseline and follow-up information and 1,256 participants with complete baseline information on blood pressure and T2D, respectively (S1 Fig).

### Statistical analyses

To take the number of participants who were lost to follow-up into account and to relate associations to the source population of 5,939 participants, most analyses (except for the linear mixed model) were weighted using an inverse probability weighting approach stratified for sex, age (10-year age groups), T2D status, and study [29]. Thus, all results refer to the weighted dataset, if not stated otherwise. Robust variance estimations appropriate to the weighting scheme were computed using the SAS procedures SURVEYMEANS and SURVEYFREQ.

Descriptive methods with means, standard deviations, confidence intervals (95% CI) and proportions were applied.

To assess the association of exposures with BP independent from potential confounding factors and over time, linear mixed models were computed for each variable of interest separately and in a second step significant co-variables were included in a basic multivariable model to examine adjusted effects. An unstructured correlation matrix was specified. SBP and DBP were approximately normally distributed, after the assessment of histograms.

Additionally, to assess whether the results hold when outlying blood pressure values were not considered, a sensitivity analysis was conducted excluding participants with baseline or follow-up SPB below the baseline 5%-quantile or above the baseline 95%-quantile (i.e. ≤108.5 or ≥177 mmHg).

We conducted additional analyses stratified by study to assess potential differences in findings by studies. Since these analyses revealed comparable findings and supported our main messages and conclusions, only the pooled results are presented.

A two-sided alpha level of 0.05 was chosen as criterion for statistical significance. All analyses were carried out using SAS, version 9.3 (SAS Institute Inc., Cary, NC, USA).

## Results

### Participants

The distributions of characteristics stratified for the three studies are presented in Table 1. About 50% of the participants were women, the mean age at baseline was 59 years and the mean age at follow-up was 65 years. At baseline, 10% had prevalent T2D –ranging from 6% in KORA to 13% in CARLA. This estimate increased to 16% in the follow-up. The frequency of hypertension across all participants defined by blood pressure measurements ≥140/90 mmHg or the intake of antihypertensive medication increased slightly from 60% at baseline to 64.5% at follow-up. Median SBP at baseline was 137 mmHg, 25% of all participants had SBP > 151 mmHg, 5% > 175 mmHg and 1% > 194 mmHg. At follow up, the median SBP had decreased to 132 mmHg– 25% of participants had SBP > 144 mmHg, 5% > 170 mmHg and 1% > 190 mmHg. Corresponding DBP measurements at baseline were 84 mmHg (median), > 91 mmHg (25%), > 103 mmHg (5%) and > 113 mmHg (1%) and 79 mmHg (median), > 86 mmHg (25%), > 98 mmHg (5%) and > 108 mmHg (1%) at follow-up.

### Degrees of awareness, treatment and control of hypertension, stratified by T2D status

Participants who reported incident T2D at the follow-up examinations were still non-diabetic (though possibly pre-diabetic or undiagnosed) at baseline, but were regarded as persons with future T2D and thus termed ‘incident T2D group’ in baseline and follow-up. Thus, 84.4% of the study participants were not affected by T2D, 5.8% had incident and 9.7% had prevalent T2D.

Participants without T2D were younger and more often female than participants with incident or prevalent T2D. As presented in Table 2, the frequency of hypertension (known or unknown) was much higher in participants with T2D than in those without T2D (85.2% vs. 56.5%) and increased by about 5% in all T2D groups from baseline to follow-up. During the same time, the degrees of awareness, treatment and control increased substantially, especially in persons with prevalent or incident T2D. The degrees of treatment reached more than 90% in people with T2D. However, even though they approximately doubled, the degrees of control remained insufficiently low at around 50%, somewhat higher in those without T2D and with incident T2D than in those with prevalent T2D.

**Table 2:** Degrees of awareness, treatment, and control at baseline and follow-up, stratified by T2D status and sex.

	Non-T2D	Non-T2D	Future incident T2D	Incident T2D	Prevalent T2D	Prevalent T2D
	^●^BL	^●●^FUP	BL	FUP	BL	FUP
**Frequency** of known or unknown hypertension						
**Total**	56.5 (55.2; 57.9)	60.3 (59.0; 61.7)	75.7 (71.2; 80.2)	81.6 (77.5; 85.7)	85.2 (82.3; 88.1)	90.3 (87.9; 92.7)
**Women**	49.5 (47.5; 51.4)	55.8 (53.9; 57.7)	73.6 (66.1; 81.1)	78.6 (71.6; 85.6)	88.6 (84.7; 92.4)	92.0 (88.8; 95.3)
**Men**	63.9 (62.0; 65.8)	65.1 (63.2; 66.9)	77.0 (71.3; 82.6)	83.5 (78.5; 88.4)	82.3 (78.1; 86.6)	88.9 (85.3; 92.3)
**Awareness** of hypertension in those with hypertension						
**Total**	66.6 (64.9; 68.4)	85.1 (83.8; 86.3)	79.8 (75.0; 84.7)	96.0 (93.7; 98.3)	83.3 (80.0; 86.6)	95.5 (93.7; 97.3)
**Women**	73.9 (71.5; 76.4)	87.9 (86.2; 89.6)	85.6 (78.7; 92.6)	98.9 (96.8; 100.0)	91.3 (87.7; 94.9)	97.8 (96.0; 99.6)
**Men**	60.7 (58.3; 63.2)	82.5 (80.7; 84.4)	76.4 (69.9; 82.9)	94.3 (90.9; 97.7)	76.0 (70.8; 81.2)	93.4 (90.5; 96.3)
**Treatment** for hypertension in those with hypertension						
**Total**	48.1 (46.3; 50.0)	73.2 (71.6; 74.8)	65.5 (59.7; 71.2)	91.6 (88.3; 94.8)	75.0 (71.2; 78.8)	91.0 (88.6; 93.5)
**Women**	55.9 (53.2; 58.7)	76.9 (74.7; 79.1)	73.7 (65.0; 82.5)	94.1 (89.6; 98.6)	84.4 (79.8; 89.0)	96.1 (93.7; 98.6)
**Men**	41.8 (39.4; 44.3)	69.9 (67.6; 72.1)	60.5 (53.1; 68.0)	90.0 (85.6; 94.4)	66.3 (60.6; 72.1)	86.5 (82.4; 90.5)
**Treatment** for hypertension in those aware of hypertension						
**Total**	72.2 (70.2; 74.2)	86.0 (84.7; 87.4)	82.0 (76.8; 87.2)	95.4 (92.9; 97.9)	90.0 (87.1; 92.9)	95.3 (93.5; 97.2)
**Women**	75.6 (72.9; 78.4)	87.5 (85.7; 89.3)	86.1 (78.7; 93.5)	95.2 (91.1; 99.3)	92.4 (88.9; 96.0)	98.3 (96.7; 99.9)
**Men**	68.9 (65.9; 71.8)	84.7 (82.7; 86.6)	79.2 (72.1; 86.3)	95.5 (92.3; 98.6)	87.3 (82.6; 92.0)	92.6 (89.4; 95.8)
**Controlled** hypertension in those with hypertension						
**Total**	17.8 (16.4; 19.2)	43.6 (41.8; 45.3)	24.6 (19.4; 29.8)	53.5 (47.7; 59.3)	23.7 (20.0; 27.5)	44.4 (40.2; 48.7)
**Women**	24.6 (22.2; 26.9)	49.9 (47.3; 52.5)	32.7 (23.4; 42.0)	56.1 (46.6; 65.6)	37.3 (31.1; 43.4)	50.1 (43.9; 56.4)
**Men**	12.4 (10.8; 14.0)	37.9 (35.5; 40.3)	19.8 (13.7; 25.9)	52.0 (44.6; 59.3)	11.2 (7.3; 15.1)	39.4 (33.6; 45.1)
**Controlled** hypertension in those aware of hypertension						
**Total**	26.7 (24.7; 28.7)	51.2 (49.3; 53.1)	30.8 (24.5; 37.0)	55.7 (49.8; 61.6)	28.5 (24.1; 32.8)	46.5 (42.2; 50.9)
**Women**	33.2 (30.2; 36.2)	56.8 (54.0; 59.5)	38.1 (27.7; 48.6)	56.7 (47.2; 66.3)	40.8 (34.3; 47.4)	51.3 (44.9; 57.6)
**Men**	20.4 (17.8; 23.0)	45.9 (43.2; 48.6)	25.9 (18.2; 33.5)	55.1 (47.6; 62.6)	14.8 (9.8; 19.7)	42.1 (36.1; 48.2)
**Controlled** hypertension in those treated for hypertension						
**Total**	37.0 (34.5; 39.6)	59.5 (57.5; 61.6)	37.5 (30.3; 44.8)	58.4 (52.4; 64.4)	31.6 (26.9; 36.4)	48.8 (44.3; 53.3)
**Women**	43.9 (40.2; 47.6)	64.9 (62.1; 67.7)	44.3 (32.8; 55.8)	59.6 (49.9; 69.3)	44.2 (37.3; 51.1)	52.1 (45.8; 58.5)
**Men**	29.6 (26.1; 33.1)	54.2 (51.3; 57.2)	32.6 (23.4; 41.9)	57.7 (50.1; 65.4)	16.9 (11.3; 22.5)	45.5 (39.2; 51.8)

*Numbers are weighted and given as % with 95% CI in brackets*.

^●^BL: Baseline

^●●^FUP: Follow-Up

Hypertension was defined as blood pressure ≥140/90 mmHg and/or intake of anti-hypertensive medication.

Irrespective of T2D status and examination time, the degrees of awareness, treatment and control in those with hypertension increased with increasing age. Control in those treated for hypertension was similar in all three age groups (data not shown).

### Trends in blood pressure parameters and hypertension control between baseline and follow-up

S1 Table shows mean SBP, DBP, and PP measurements stratified for T2D status and baseline / follow-up as well as calculated differences between the baseline and follow-up pairs respectively. The mean SBP increased from non-T2D (137.09 mmHg) to prevalent T2D (148.52 mmHg) and decreased consistently in all T2D groups from baseline to follow-up, antidromic to the usual increase of SBP with increasing age. The greatest reduction was found in incident T2D with -8.55 mmHg. The DBP was similar in all groups and decreased from 84.38 mmHg to 77.28 mmHg in prevalent T2D. The PP was markedly higher in persons with T2D than in persons without T2D (64.14 mmHg in prevalent T2D compared to 52.87 mmHg in non-T2D) and did not change much between the two examinations.

**Fig 1:**
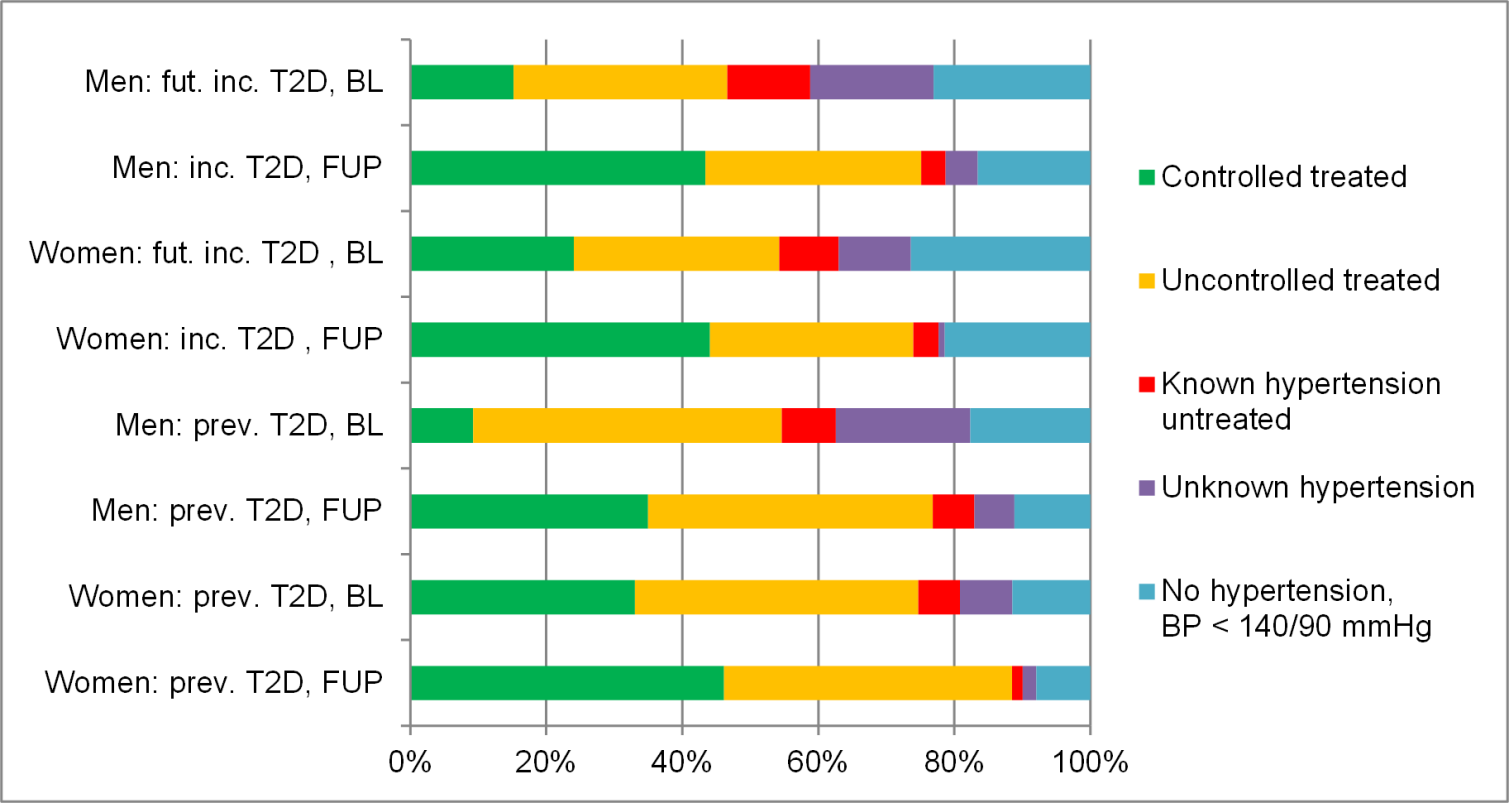
Sex-stratified frequencies of controlled treated, uncontrolled, known but untreated and unknown hypertension in participants with prevalent and (future) incident T2D in the baseline and follow-up examinations. Hypertension was defined as blood pressure ≥140/90 mmHg. *Numbers are weighted and given as %*.

Trends of blood pressure treatment and control are illustrated in S2 Fig and Fig 1: In the baseline examinations, a much greater percentage of participants without T2D was free of hypertension than of those with T2D (42% vs. 15%). Compared to baseline, the percentage of participants with high blood pressure increased slightly in participants without T2D, the percentage with controlled treated hypertension more than doubled (from 10 to 26%), while the number of persons with insufficiently treated hypertension approximately stayed the same (18%). Both untreated known and untreated unknown hypertension became less frequent.

In participants with prevalent T2D, the percentage without hypertension decreased as well, while the number of controlled treated persons increased greatly from 20 to 40%. Insufficiently treated hypertension remained similar at about 43%. As in people without T2D, the frequencies of untreated and unknown hypertension became rare (each about 4%).

Finally, participants with incident T2D as reported in the follow-up examinations stood between people without and people with prevalent T2D: in follow-up 18% were not affected with hypertension, 44% were controlled treated, 31% were insufficiently treated, 4% had untreated and 3% unknown hypertension. Thus, the number of people affected with hypertension has increased in all subgroups with time. The frequencies of controlled treated hypertension increased markedly in all T2D groups, the frequencies of insufficiently treated hypertension remained stable and the percentages of untreated and unknown cases decreased consistently.

Fig 1 presents analogous percentages in people with incident or prevalent T2D stratified by sex. Hence, the estimate of men with controlled hypertension has caught up on the percentage of women in follow-up. Persons with incident T2D were still not as often affected by hypertension as people with prevalent T2D. Treatment and control were similar in incident and prevalent T2D and the number of persons with unknown hypertension was small, especially in women.

### Hypertension and concomitant high pulse pressure by T2D status and age

With increasing age and T2D status, the percentages of participants with an unfavorable combination of both uncontrolled blood pressure (≥140/90 mmHg) and high PP (≥60 mmHg) in follow-up increased consistently. In participants without T2D, aged 45 to 54 years, 9% had both conditions, compared to 17% of participants with prevalent T2D in the same age group. In the age group of 75 to 83 years this number increased to 31% in people without and 55% in people with prevalent T2D. Incident T2D stood in between in most age groups (S3 Fig).

Means and 95% CIs of SBP in baseline and follow-up, stratified by sex and T2D status are depicted in Table 3. SBP was generally higher in men than in women. The respective means in each T2D subgroup decreased considerably more in men than in women from baseline to follow-up (e.g. -9.2 mmHg vs. –7.5 mmHg in people with incident T2D) however, mean readings in follow-up were still clearly higher in men then in women.

**Table 3:** Descriptive changes in systolic blood pressure stratified by sex and T2D status.

**Men (50.2%)**	**Total**	**Non-T2D**	**Incident T2D**	**Prevalent T2D**
**%**	**100.0**	**82.4**	**7.2**	**10.4**
Mean SBP,^●^BL	142.84	141.33	146.30	152.42
95% CI	(142.03; 143.65)	(140.48; 142.19)	(143.25; 149.34)	(149.42; 155.41)
Mean SBP, ^●●^FUP	136.40	135.49	137.10	143.04
95% CI	(135.58; 137.21)	(134.63; 136.36)	(133.63; 140.56)	(140.09; 145.98)
Difference (FUP-BL)	-6.45	-5.84	-9.20	-9.38
95% CI	(-7.29; -5.61)	(-6.72; -4.95)	(-12.80; -5.61)	(-12.50; -6.26)
**Women (49.8%)**				
**%**	**100.0**	**86.5**	**4.5**	**9.0**
Mean SBP, BL	134.31	133.01	139.81	143.99
95% CI	(133.48; 135.14)	(132.16; 133.87)	(135.79; 143.85)	(140.86; 147.12)
Mean SBP, FUP	129.97	128.82	132.30	139.76
95% CI	(129.14; 130.79)	(127.97; 129.68)	(128.55; 136.05)	(136.40; 143.13)
Difference (FUP-BL)	-4.34	-4.19	-7.52	-4.23
95% CI	(-5.14; -3.55)	(-5.00; -3.28)	(-12.03; -3.11)	(-7.80; -0.66)

^●^BL: Baseline

^●●^FUP: Follow-Up

### Adjusted analyses including basic personal attributes associated with SBP and change in SBP: Linear mixed models

Adjusted linear mixed models confirmed this association and generated further insights (Table 4): Including observation time, sex, age at baseline, study, T2D at follow-up and interaction terms of each variable with follow-up time, we found that SBP was significantly lower in KORA than in SHIP and CARLA and participants of KORA and CARLA had greater reductions of SBP from baseline to follow-up. Compared to the youngest age group (45–54 years) SBP was 3 mmHg higher in persons aged 55–64 and 9 mmHg higher in persons aged 65–74. At follow-up, however, SBP had decreased more in the oldest than the youngest and middle-aged groups (-3 mmHg). People with prevalent T2D had highest SBP readings (+8 mmHg), followed by people with incident T2D (+5 mmHg) as compared to those without T2D. These values decreased most in incident T2D (-3 mmHg).

**Table 4:** Linear mixed model including observation time, sex, BL-age, study, FUP-T2D and interaction terms with time as covariates and systolic BP as outcome (N = 4683, complete case dataset).

	ß	p-value
**Intercept**	133.82	< .0001
**Time** ^●^BL (ref.)	0	
Time ^●●^FUP	-3.86	< .0001
**Observation time/yearMean time 5.6 years**	1.317.34	0.0307
**Sex** Women	-7.94	< .0001
**Sex** Men (ref.)	0	
Women*FUP	1.80	0.0017
**Study** CARLA	1.71	0.0871
**Study** KORA	-10.38	< .0001
**Study** SHIP	0	
CARLA*FUP	-1.98	0.0073
KORA*FUP	-2.77	< .0001
**BL-Age** 45–54 (ref.)	0	
**BL-Age** 55–64	3.42	< .0001
**BL-Age** 65–74	8.94	< .0001
55–64*FUP	1.29	0.0536
65–74*FUP	-3.22	< .0001
**T2D** No (ref.)	0	
**T2D** Incident	4.91	< .0001
**T2D** Prevalent	7.81	< .0001
Incident T2D*FUP	-3.12	0.0115
Prevalent T2D*FUP	-1.83	0.0797

*Estimates are not weighted*

^●^BL: Baseline

^●●^FUP: Follow-Up

Using effect estimators of the model we calculated two exemplary profiles: A woman, aged 45–54 years, with an observation time of 5.6 years, who participated in KORA and did not have T2D would have a SPB of 118 mmHg at follow-up. In contrast, a man in the oldest age group, with an observation time of 5.6 years, who participated in SHIP and had prevalent T2D would have a SBP of 149 mmHg at follow-up.

### Exemplary FUP-SBP calculations with mean observation time of 5.6 years

#### Young KORA woman without T2D

133.82 (intercept) – 3.86 (FUP) +7.34 (mean observation time) - 7.94 (woman) + 1.80 (woman change) – 10.38 (KORA) – 2.77 (KORA change) + 0 (age 45–54) + 0 (no T2D) = **118.01 mmHg**

#### Elderly SHIP man with prevalent T2D

133.82 (intercept) – 3.86 (FUP) + 7.34 (mean observation time) + 0 (man) + 0 (SHIP) + 8.94 (age 65–74) – 3.22 (65–74 change) + 7.81 (prevalent T2D) – 1.83 (prevalent T2D change) = **149.00 mmHg**

### Sensitivity analyses to regarding outlying values

In a sensitivity analysis, we excluded participants with baseline or follow-up SPB below the baseline 5%-quantile or above the baseline 95%-quantile (i.e. ≤108.5 or ≥177 mmHg, 17.1%) to assess possible effects of outlying blood pressure values. Since the results of descriptive analyses and the adjusted linear mixed model remained similar (data not shown), we assume that regression-to-the-mean played a minor role in our study.

### Numbers and percentages of anti-hypertensive drugs

In the subgroup of participants with a physician’s diagnosis of hypertension and anti-hypertensive treatment both at baseline and follow-up (Table 5), one to eight different preparations and one to eight active agents were used. Combinations of different active agents in one preparation are very common in Germany (taking only one pill instead of two or more is considered to improve patient adherence).

**Table 5:** Numbers of preparations and active agents and percentages of anti-hypertensive medications groups.

	Non-T2D	Non-T2D	Future incident T2D	Incident T2D	Prevalent T2D	Prevalent T2D
	^●^BL	^●●^FUP	BL	FUP	BL	FUP
**number of preparations** *	1.6 (1.5; 1.6)	2.0 (1.9; 2.0)	1.7 (1.6; 1.9)	2.1 (2.0; 2.3)	2.0 (1.8; 2.1)	2.4 (2.2; 2.5)
**number of diff. active components**	1.9 (1.8; 2.0)	2.4 (2.3; 2.5)	2.1 (1.9; 2.3)	2.7 (2.5; 2.9)	2.4 (2.2; 2.5)	2.9 (2.8; 3.1)
** **% beta blocking agents**	54.2 (51.5; 57.0)	63.6 (61.0; 66.3)	49.9 (42.3; 57.5)	62.7 (55.3; 70.0)	47.8 (42.7; 53.0)	65.7 (60.8; 70.6)
**% ACE inhibitors**	39.4 (36.7; 42.1)	44.6 (41.8; 47.3)	46.7 (39.2; 54.3)	60.6 (53.2; 68.0)	61.8 (56.8; 66.8)	64.9 (60.0; 69.8)
**% diuretics**	36.7 (34.1; 39.3)	49.3 (46.6; 52.1)	43.0 (35.5; 50.6)	59.2 (51.7; 66.6)	48.1 (43.0; 53.3)	62.9 (57.9; 67.9)
**% angiotensin antagonists**	16.0 (14.0; 18.0)	29.9 (27.4; 32.4)	10.6 (05.9; 15.3)	23.2 (16.8; 29.6)	13.1 (09.6; 16.5)	23.1 (18.7; 27.4)
**% calcium channel blockers**	31.6 (29.1; 34.2)	38.5 (35.8; 41.2)	37.4 (30.0; 44.7)	35.9 (28.6; 43.2)	40.5 (35.4; 45.6)	46.0 (40.8; 51.1)
**% other anti-hypertensives**	5.7 (4.4; 7.0)	5.3 (4.1; 6.5)	9.7 (5.2; 14.3)	6.8 (3.0; 10.6)	12.8 (9.4; 16.3)	12.7 (9.3; 16.2)

*Numbers are weighted and given with 95% CI*.

^●^BL: Baseline

^●●^FUP: Follow-Up

*including combinations of different active agents

**percentage of study participants who used at least one active component from this group

The mean number of preparations and active components used increased from baseline to follow-up in all T2D groups (except for the ATC group “other anti-hypertensives”). Participants with prevalent T2D used more drugs than participants without T2D or with incident T2D. At follow-up most people still used the same drug group(s) they had used at baseline, but a considerable percentage changed (data not shown).

## Discussion

### Key results

Using data of the German DIAB-CORE Consortium, we found that blood pressure management has developed positively during past years. At baseline and follow-up hypertension prevalence, awareness and treatment were considerably higher in participants with T2D than in those without T2D. While awareness and treatment improved clearly between baseline and follow-up, especially in those with T2D (reaching more than 90%), hypertension control was achieved only in about half the number of people irrespective of T2D status. In contrast to the known elevation of SBP with advancing age, SBP decreased in all subgroups by 5.0 to 8.6 mmHg. However, the percentages of people with uncontrolled high blood pressure were still considerable, especially in the older age groups. Persons with incident or prevalent T2D were still often insufficiently treated. For example, more than 35% of those aged 55 to 64 years in follow-up with prevalent T2D had uncontrolled blood pressure ≥140/90 mmHg and concomitant PP ≥60 mmHg. Fortunately, the percentages of participants with unknown or known but untreated hypertension have become very small. The linear mixed model figured out that blood pressure in men improved more than in women at follow-up, however, men still had higher mean readings than women. The KORA study had more favourable results than the other studies. SBP decreased more in the oldest than in the youngest and middle-aged groups. People with prevalent T2D had highest SBP, followed by people with incident T2D and those without T2D. At follow-up, SBP had decreased most in those with incident T2D. These results indicate more intensive treatment in hitherto more problematic groups, namely in men, older people and those with newly diagnosed diabetes.

### Generalization

Some German and various international studies have analysed trends in hypertension control in people with and/or without T2D which were in line with our results. For Germany, a recently published study by Neuhauser et.al [30] used data of 7,108 adult participants of the German Health Interview and Examination Survey (GNHIES98, 1998) as baseline and 7,095 participants of the German Health Interview and Examination Survey for Adults (DEGS1, 2008–2011) as follow-up study. The mean systolic blood pressure in GNHIES98 was lower than in our study (e.g. women aged 45–64 years: 131.3 vs. 131.6 mmHg, men aged 45–64 years: 133.2 vs. 140.9 mmHg). An improvement of awareness, treatment and control was reported between GNHIES98 and DEGS1. Blood pressure control increased at an order of magnitude, which is well comparable to DIAB-CARE: among GNHIES98/DEGS1 participants hypertension control increased from 23% to 51% and among all DIAB-CARE participants regardless of T2D status from 19% to 44%. Degrees of treatment and awareness were also similar. In our study 76% of all participants who were aware of having hypertension used at least one antihypertensive drug at baseline and 88% at follow-up compared to 79% and 88% in GNHIES98/DEGS1, which is also very well comparable. Moreover, GNHIES98/DEGS1 found more beneficial readings in women than in men as we did in our study.

Ott et al. (2009) [31] conducted a prospective 4-year follow-up study in 2,914 patients with T2D aged 35–80 years in daily practice and found very little improvement from 2002 to 2007. Mean blood pressure decreased from 139.3/80.0 mmHg at BL baseline to 137.3/79.9 mmHg at follow-up and anti-hypertensive medication was prescribed more often. Labeit et al. (2012) [32] compared 55,518 primary care patients with and without T2D that were recruited in 2003 in the course of the DETECT study, with the HYDRA study which was conducted in 2001. The estimates of treated and controlled hypertension remained low at about 56% and 20% with some minor improvement. Using three surveys of the KORA study conducted between 1999 and 2008, Schunk et al. found clearer enhancements in blood pressure control in participants with T2D: the proportions of people who achieved the goal blood pressure of < 140/90 mmHg were 43.6%, 55.2%, and 70.5% across the three studies sorted by examination dates [33].

Furthermore, results of the KORA study from 2008 indicated that participants of disease management programs (DMPs) for people with T2D that were launched in 2002, reached the same treatment goal significantly more often (79.8% vs. 63.6%, p-value: 0.037) than controls [34].

Finally, international findings are consistent: longitudinal studies on hypertension trends from the Netherlands (1998–2004) [35], Spain (2003–2009) [36], the US (1988–2008 and 2001–2010) [37, 38], and China (1982–2010) [39] consentaneously concluded that hypertension management has improved, but remains insufficient and in need for further monitoring and enhancement.

### Strengths and Limitations

Our dataset included three population-based studies with well comparable questionnaires and measurements. Nevertheless, we cannot exclude systematic divergences due to slightly different designs, protocols or measuring devices. This is also true for the comparability of baseline and follow-up examinations as conditions may change. However, the improvement of mean blood pressure control was consistent in all studies though and thus appears very plausible.

Using longitudinal data, we were able to track changes between baseline and follow-up readings from each individual participant and draw conclusions to a general improvement of blood pressure control over time. However, we cannot rule out that a potential bias due to loss to follow-up may have influenced our findings. To compensate for this missing follow-up information, we applied an inverse probability weighting approach which is aimed to reduce a potential bias [29].

The observation times were different across and within studies. This was accounted for in the adjusted models, but not considered in the descriptive results.

By applying inverse probability weighting, we intended to improve the internal quality of our study. However, we do not claim representativeness of our data regarding the population of Germany.

As in our previous publications on blood pressure [23, 24], we could not identify participants with “white coat hypertension”, who may be represented by a part of those with unknown, uncontrolled or insufficiently controlled hypertension. This form of hypertension occurs in the special setting of a physician’s office and might also have affected participants in the study centres due to the unfamiliar situation. In an untreated Finnish population, the prevalence of white coat hypertension was about 15% and risk factors included lower BMI and non-smoking status [40]. Taking three measurements and the mean of the second and third reading for our analyses might have diminished this phenomenon.

Finally, the participants received letters with their diagnostic findings subsequent to the examinations at the study centres and were asked to consult a physician if they had elevated blood pressure measurements. This information might have induced some improvement in hypertension control, but we do not assume that the bigger part of our results is due to this one-time intervention effect.

### Conclusion and prospect

Our pooled analyses including three regional population-based studies intended to contribute information on the development of health care regarding hypertension in people with and without T2D in Germany. In accordance with other German and international investigations, we found that the degrees of awareness, treatment and control increased considerably, but nevertheless remained insufficient. Even though more than 90% of people with T2D and hypertension are aware and treated for their high blood pressure, target values are only reached by about 50%. This number is even lower than in people without T2D (with about 60%), indicating that treatment regimens, agents, combinations etc. are not yet optimally adjusted, especially in diabetes care. Although a great number of different anti-hypertensive medications has been approved and is currently in use, successful treatment of hypertension is not a trivial subject. In the light of its outstanding importance for health in developed and developing countries, research on primary prevention and new treatment options is still in great demand.

## Supporting Information

S1 Fig Flowchart. (TIF)

S2 Fig Frequencies of controlled treated, uncontrolled, known but untreated and unknown hypertension in participants without, with prevalent and with incident T2D in the baseline and follow-up examinations. Hypertension was defined as blood pressure ≥140/90 mmHg, N = 5,939, weighted dataset.
(TIF)

S3 Fig Percentages of participants with uncontrolled blood pressure ≥140/90 mmHg and concomitant pulse pressure values ≥60 mmHg, stratified by T2D and age groups in the follow-up examinations. (TIF)

S1 Table Blood pressure parameters at baseline and follow-up, stratified by T2D status. *Numbers are weighted and given as means with 95% CI in brackets*, *SBP: Systolic blood pressure. **DBP: Diastolic blood pressure. ***PP: Pulse pressure. ^●^BL: Baseline. ^●●^FUP: Follow-Up.
(DOC)
