# Supplementary figures and images for: Blood Pressure Control Has Improved in People with and without Type 2 Diabetes but Remains Suboptimal: A Longitudinal Study Based on the German DIAB-CORE Consortium
Source: PLoS One. 2015 Jul 29;10(7):e0133493. doi: 10.1371/journal.pone.0133493 (PMC4519307; doi:10.1371/journal.pone.0133493)

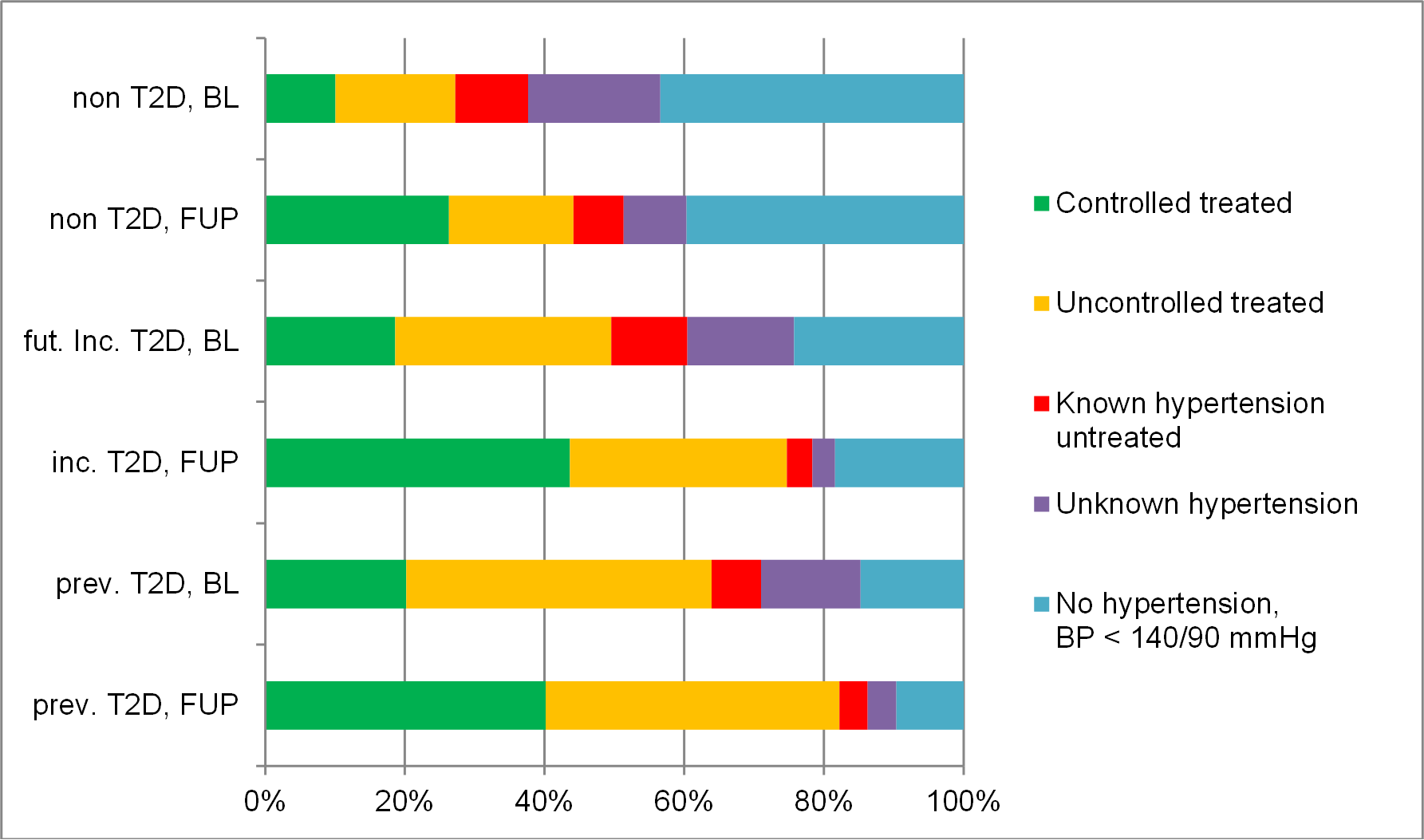

Supplement: S2 Fig — Hypertension was defined as blood pressure ≥140/90 mmHg, N = 5,939, weighted dataset. (TIF) [file pone.0133493.s002.tif]

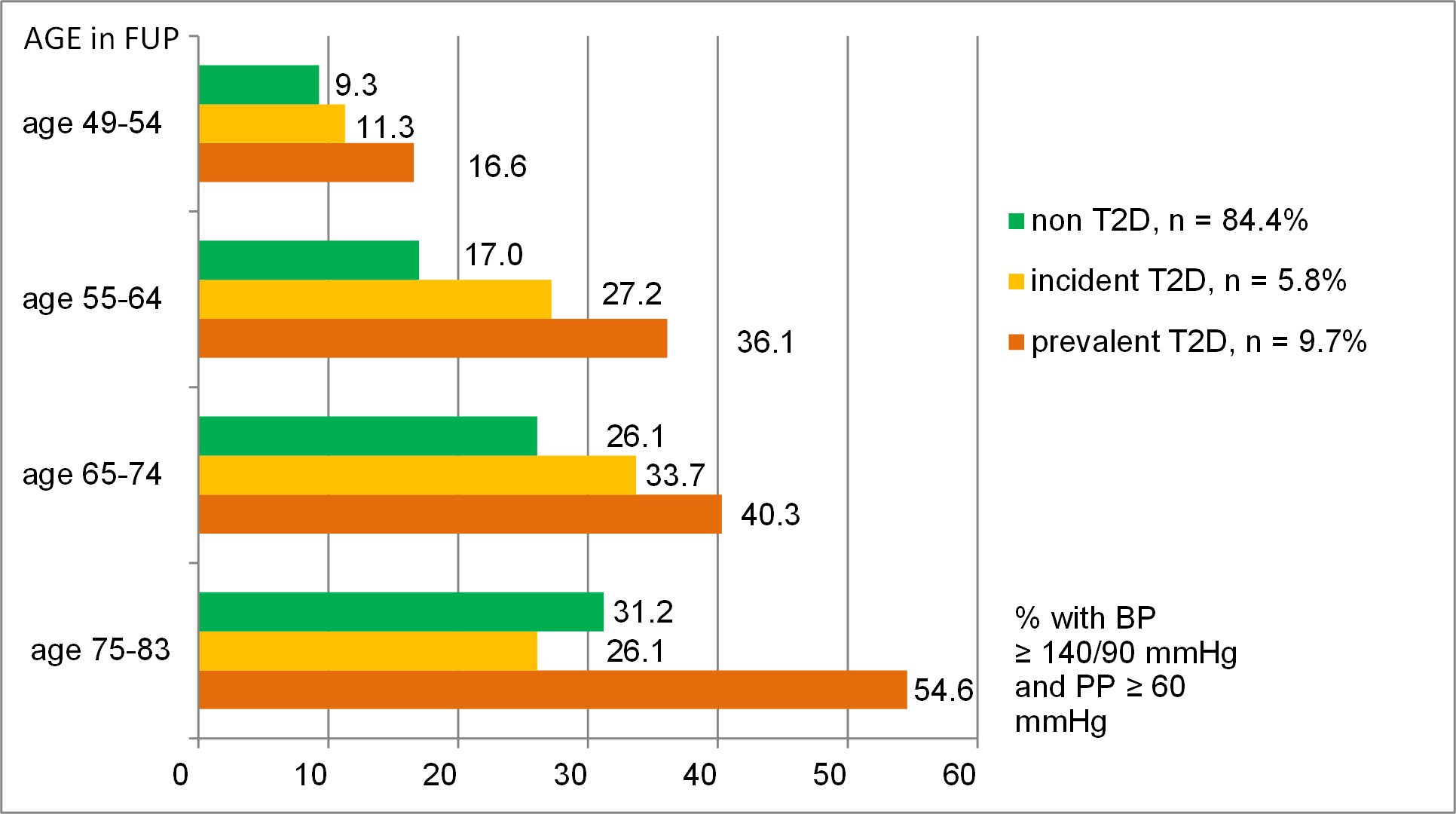

Supplement: S3 Fig — (TIF) [file pone.0133493.s003.tif]
