# Supplementary material for: Blood Pressure Control Has Improved in People with and without Type 2 Diabetes but Remains Suboptimal: A Longitudinal Study Based on the German DIAB-CORE Consortium
Source: PLoS One. 2015 Jul 29;10(7):e0133493. doi: 10.1371/journal.pone.0133493 (PMC4519307; doi:10.1371/journal.pone.0133493)
Supplement: S1 Table — Numbers are weighted and given as means with 95% CI in brackets, *SBP: Systolic blood pressure. **DBP: Diastolic blood pressure. ***PP: Pulse pressure. ●BL: Baseline. ●●FUP: Follow-Up. (DOC) [file pone.0133493.s004.doc]

**S1_Table: Blood pressure parameters at baseline** and follow-up, stratified by T2D status.

|  | **Non-T2D** | **(Future)**  **Incident T2D** | **Prevalent T2D** |
| --- | --- | --- | --- |
| **Baseline** |  |  |  |
| **%** | **84.4** | **5.8** | **9.7** |
| **Age** mean | 58.56 | 60.98 | 62.87 |
| (95% CI) | (58.30; 58.82) | (60.01; 61.94) | (62.14; 63.59) |
| **Women** (%) | 51.0 | 38.3 | 46.2 |
| ***SBP** (mmHg) | **137.09** | **143.81** | **148.52** |
| mean (95% CI) | (136.47; 137.71) | (141.37; 146.26) | (146.34; 150.71) |
| ****DBP** (mmHg) | **84.22** | **85.58** | **84.38** |
| mean (95% CI) | (83.88; 84.56) | (84.23; 86.94) | (83.25; 85.52) |
| *****PP** (mmHg) | **52.87** | **58.23** | **64.14** |
| mean (95% CI) | (52.44; 53.30) | (56.40; 60.07) | (62.41; 65.87) |
| **Follow-up** |  |  |  |
| **SBP** | **132.09** | **135.26** | **141.52** |
| mean (95% CI) | (131.47; 132.71) | (132.69; 137.83) | (139.31; 143.74) |
| **DBP** | **79.26** | **77.79** | **77.28** |
| mean (95% CI) | (78.92; 79.60) | (76.45; 79.12) | (76.14; 78.41) |
| **PP** | **52.83** | **57.47** | **64.25** |
| mean (95% CI) | (52.40; 53.27) | (55.59; 59.35) | (62.48; 66.02) |
| **Differences** |  |  |  |
| ^●●^**FUP (SBP) -** ^●^**BL (SBP)** | **-5.00** | **-8.55** | **-7.00** |
| (95% CI) | (-5.60; -4.40) | (-11.35; -5.76) | (-9.35; -4.64) |
| (change in % of BL) | (-3.6) | (-6.0) | (-4.7) |
| **FUP(DBP) - BL(DBP)** | **-4.96** | **-7.79** | **-7.11** |
| (95% CI) | (-5.28; -4.64) | (-9.25; -6.33) | (-8.31; -5.90) |
| (change in % of BL) | (-5.9) | (-9.1) | (-8.4) |
| **FUP(PP) - BL(PP)** | **-0.04** | **-0.76** | **0.11** |
| (95% CI) | (-0.43; 0.36) | (-2.66; 1.14) | (-1.53; 1.75) |
| (change in % of BL) | (-0.1) | (-1.3) | (+0.2) |

*Numbers are weighted and given as means with 95% CI in brackets,*

*SBP: Systolic blood pressure

**DBP: Diastolic blood pressure

***PP: Pulse pressure

^●^BL: Baseline

^●●^FUP: Follow-Up
